# A novel technique for automatic hybrid modulation classification in UWA communications using 3D constellation diagrams

**DOI:** 10.1038/s41598-026-64630-5

**Published:** 2026-08-14

**Authors:** Mohamed A. Abdel-Moneim, Khalil F. Ramadan, El-Sayed M. El-Rabaie, Fathi E. Abd El-Samie, Nariman Abdel-Salam

**Affiliations:** 1 Department of Telecommunication, Faculty of Engineering, Egyptian Russian University, Cairo, 11829 Egypt; 2 Department of Electronics and Electrical Communications Engineering, Faculty of Electronic Engineering, Menoufia University, Menouf, 32952 Egypt; 3 Electrical Communication and Electronic Systems Engineering Department, Faculty of Engineering, October University for Modern Sciences and Arts (MSA), 6th of October City, Egypt

**Keywords:** Automatic modulation classification (AMC), Hybrid modulation classification, Underwater acoustic (UWA) communication, Three-dimensional (3D) constellation diagram, Engineering, Mathematics and computing

## Abstract

Underwater acoustic (UWA) communication systems operate in severe channel impairments, including strong multipath propagation, long delay spreads, frequency selectivity, Doppler effects, and high ambient noise. These challenges significantly complicate automatic modulation classification (AMC), especially in dense underwater networks where interference further degrades performance. In this paper, we investigate AMC for frequency-indexed three-dimensional hybrid modulation schemes, namely frequency-phase keying (FPK) and frequency quadrature amplitude modulation (FQAM), in UWA environments. While AMC has been extensively studied for conventional modulation formats, its application to frequency-indexed hybrid modulation schemes in UWA communication systems has received comparatively limited attention in the existing literature. The proposed approach depends on a three-dimensional (3D) constellation representation for robust feature extraction and modulation discrimination. The resulting 3D signal representations are converted into image-based inputs and processed with deep convolutional neural network (CNN) models, including AlexNet, VGG-19, and ResNet50, to automatically extract discriminative features and enable reliable classification. The proposed framework is evaluated under both single-carrier (SC) and orthogonal frequency-division multiplexing (OFDM) transmission schemes to comprehensively assess its robustness and adaptability. Simulation results demonstrate accurate modulation recognition under severe UWA conditions and low signal-to-noise ratio (SNR), confirming the effectiveness of combining hybrid modulation with deep-learning-based AMC for next-generation UWA communication systems.

## Introduction

AMC is essential in UWA communication systems for applications such as interference detection and spectrum monitoring^1^. With the growing deployment of submarines and autonomous underwater vehicles (AUVs), the demand for reliable AMC solutions continues to rise. As a critical stage between signal detection and demodulation, AMC plays a central role in the signal processing chain. However, implementing AMC in UWA environments is particularly challenging due to severe multipath propagation, long delay spreads, and Doppler effects^2^. Traditional AMC methods are generally categorized into likelihood-based (LB) and feature-based (FB) methods. The LB methods depend on estimating the likelihood function for each modulation scheme under the assumption of perfect channel state information^3^. However, a typical UWA channel exhibits severe multipath propagation with hundreds of channel taps, making this approach computationally demanding. In contrast, FB methods focus on extracting a limited set of representative features for classification rather than directly evaluating the likelihood function, which makes them more practical for UWA environments. These methods generally consist of two stages: feature extraction and classification. In the feature extraction stage, commonly-used features include higher-order cumulants (HOCs)^4^, cyclostationary features^5^, and wavelet-based features. In the classification stage, popular classifiers such as k-nearest neighbours (k-NN)^6^ and support vector machines (SVM)^7^ are typically employed. In recent years, deep learning (DL) techniques have emerged as powerful alternatives, enabling the extraction of more robust and discriminative features, while reducing reliance on manual feature engineering and domain expertise^8–10^.

In parallel, three-dimensional hybrid modulation techniques have attracted growing attention for UWA systems^11^. These techniques combine multiple modulation mechanisms, such as phase, amplitude, and frequency modulation, yielding a three-dimensional signal-space representation. Unlike conventional two-dimensional schemes limited to in-phase (I) and quadrature (Q) components, three-dimensional modulation exploits an additional independent dimension. Hybrid modulation denotes the functional integration of different modulation mechanisms, whereas three-dimensional modulation describes the geometric structure of the resulting signal constellation. This paper focuses on schemes that satisfy both properties.

The main contributions of this paper are as follows:

- This work investigates AMC for frequency-indexed three-dimensional hybrid modulation schemes (FPK and FQAM) in UWA communication systems, an area that has received limited attention in the existing literature.
- It introduces a 3D constellation representation that jointly leverages the in-phase (I), quadrature (Q), and frequency dimensions, thereby providing enhanced geometric separability for DL–based classification.
- The proposed approach transforms these 3D representations into image-based inputs for deep CNN models (AlexNet, VGG-19, and ResNet50), enabling robust modulation recognition under severe UWA channel conditions.
- The proposed AMC framework is investigated using both SC and OFDM transmission configurations.

The paper is structured as follows. Section "Related work" provides a concise review of related work, and Sect. "FPK and FQAM modulation" presents the FPK and FQAM modulation techniques. Section "Experimental setup" describes the experimental setup. Section "Simulation results and comparative analysis" presents the simulation results and the comparative analysis. Finally, Sect. "Conclusion" gives the conclusion of the study and outlines future research directions.

## Related work

This section provides a concise review of recent studies on CNN-based AMC methods for UWA channels, as summarized in Table 1. Li and Zha^12^ proposed a DL framework for UWA modulation classification using IQ eye-diagram representations. The method converts IQ signals into binary and grayscale eye-diagram images to preserve temporal and statistical signal characteristics before applying CNN-based feature extraction. Experimental results showed 100% classification accuracy at 10 dB SNR, and approximately 80% at 0 dB SNR, and the approach was robust to frequency-offset effects.

**Table 1:** Summary of CNN-based AMC methods.

Refs	Year	Network Architecture	Modulation Types	SNR Range (dB)	Key Contribution	Classification Performance
Li and Zha^12^	2019	CNN (Residual Learning)	BPSK, QPSK, OQPSK, 8PSK, 16QAM, 16APSK, 32APSK, 64QAM	0 to 10	IQ eye-diagram representation for CNN-based AMC	100% at 10 dB, 80% at 0 dB
Wang et al.^13^	2021	VGG-inspired CNN	AM, 2ASK, FM, 2FSK, 4FSK, BPSK, QPSK	− 6 to 14	Image-based representation of amplitude, frequency, and phase features	96.6% at 14 dB, 64.6% at 6 dB
An et al.^14^	2021	ST-CLDNN	Multicarrier waveforms (OFDM, SC-FDE, FBMC, UFMC, GFDM)	− 18 to 20	Spatial–temporal learning framework that jointly exploits I/Q and amplitude representations for blind multicarrier waveform recognition under time-varying multipath channels	99.5% at 20 dB, 70% at − 4 dB
An et al.^15^	2022	Two-stage FOC-FCNN + P-ACVNet (TCN)	BPSK, QPSK, 8PSK, 4PAM, 8PAM, 16QAM, 32QAM, 64QAM, 128QAM, 256QAM, 512QAM, 1024QAM, 2048QAM	− 10 to 30	Two-stage high-order modulation recognition based on fourth-order cumulants and projected accumulated constellation vectors (P-ACV) for OSTBC-OFDM systems	97.37% at 12 dB, ≈48% at − 10 dB
Wang et al.^16^	2022	CNN	2FSK, 4FSK, 8FSK, BPSK, LFM, OFDM	0 to 20	Multi-hydrophone feature fusion network (MHFNet)	98% at 20 dB, 75% at 0 dB
Hu et al.^17^	2023	PTR-AE and CNN	2FSK, 4FSK, 8FSK, BPSK, QPSK, OFDM	0 to 10	Passive time-reversal enhancement for multipath mitigation	> 90% at high SNR, > 70% at low SNR
Lyu et al.^18^	2024	CNN and Transformer	BPSK, QPSK, 8PSK, 4PAM, 16QAM, 64QAM, FM, DSB, CPFSK, 4FSK	− 20 to 18	Spatial–temporal feature extraction with attention mechanism	> 90% at 20 dB, ~ 10% at -20 dB
Xu and Zhang^19^	2024	Multi-task CNN	PSK, QPSK, 8PSK, QFSK, 8FSK, 16APSK, 16QAM, 64QAM, 4PAM, LFM, DSB SC, SSB SC	− 10 to 20	Joint AMC and DoA estimation	93–97% (high SNR), 85–90% (low SNR)
Jing et al.^20^	2024	CNN and MAML	BPSK, QPSK, 8PSK	8 to 15	OTFS-based AMC with meta-learning adaptation	80–90% (high SNR), 63–76% (low SNR)
Li et al.^21^	2024	SSD-MobileNet-V2-FPNLite	CW, DSSS, 2FSK, 4FSK, LFM, OFDM, BPSK, QPSK	− 10 to 10	Time–frequency image-based signal recognition	94.2% at 10 dB, 89% at -10 dB
Zhou et al.^22^	2025	DFRT (ResNet and Transformer)	2FSK, 4FSK, 2PSK, 4PSK, OFDM, DSSS, 4DSSS, 9DSSS	Distance-dependent evaluation	Dual-feature fusion using SPWVD and higher-order statistics	98.9% (high SNR), 95.8% (low SNR)

Wang et al.^13^ introduced a hybrid DL approach for modulation classification in short-burst UWA communication systems. The framework extracts amplitude, frequency, and phase characteristics from underwater signals and converts them into image-based features, which are then processed by a multilayer CNN. Experiments demonstrated 96.6% classification accuracy at 14 dB SNR and 64.6% at 6 dB SNR under harsh underwater channel conditions.

An et al.^14^ proposed a blind multicarrier waveform recognition framework based on a Spatial Temporal-Convolutional Long Short-Term Deep Neural Network (ST-CLDNN), which jointly exploits I/Q and amplitude signal representations to extract spatial and temporal features under time-varying multipath fading channels. The presented method achieved approximately 99.5% recognition accuracy at 20 dB SNR and about 70% at − 4 dB SNR, demonstrating robust performance under severe multipath and low-SNR conditions.

An et al.^15^ proposed a two-stage framework for high-order modulation recognition in non-cooperative B5G OSTBC-OFDM systems by combining fourth-order cumulants and projected accumulated constellation vector (P-ACV) features. Blind zero-forcing equalization was employed to reconstruct the received signals, while a temporal convolutional network (TCN) classified the P-ACV features. The presented method achieved approximately 97.37% accuracy at 12 dB SNR and about 48% at − 10 dB SNR, while reducing the inference time by six times compared with the suboptimal method.

Wang et al.^16^ proposed the multi-hydrophone fusion network (MHFNet) for underwater modulation classification. The framework uses multiple hydrophones and adaptive CNN-based feature fusion to improve robustness to channel fluctuations and interference. Experimental results showed nearly 98% accuracy at 20 dB SNR and about 75% at 0 dB SNR, outperforming conventional fusion-based AMC methods.

Hu et al.^17^ developed a passive time-reversal modulation classification framework to mitigate severe multipath effects in underwater channels. The approach uses a passive time-reversal autoencoder (PTR-AE) to enhance frequency-domain features before applying CNN-based classification. Experimental results showed classification accuracies above 90% at high SNR and above 70% under low-SNR conditions.

Lyu et al.^18^ proposed a hybrid spatio-temporal neural network architecture for AMC in UWA communication systems. To overcome the limitations of conventional CNNs, which treat all spatial positions uniformly, the authors incorporated a transformer-based attention mechanism. This module adaptively integrates spatial and temporal features, enabling more informative representations to be extracted directly from raw in-phase and quadrature (IQ) signal sequences, without relying on traditional constellation diagrams or eye-pattern visualizations. The presented system was evaluated across various SNR conditions, achieving over 90% classification accuracy at moderate-to-high SNR levels (20 dB) and maintaining approximately 10% accuracy at very low SNR (-20 dB), demonstrating robustness in harsh underwater environments.

Xu and Zhang^19^ developed a multi-task CNN framework that simultaneously performs modulation classification and direction-of-arrival (DoA) estimation for UWA signals. The model was trained on a large simulated dataset of 450,120 samples covering 12 modulation formats and 31 SNR levels from –10 dB to 20 dB, as well as real-world data collected from experiments in Songhua Lake. The architecture consists of an input layer followed by several residual blocks, leading to two separate output branches: one for modulation recognition and the other for DoA estimation. Simulation results indicate strong performance, with classification accuracies between 93% and 97% at high SNRs and between 85% and 90% at low SNRs. These findings highlight the model strong generalization capability across varying acoustic conditions and signal qualities.

Lianyou Jing et al.^20^ presented a CNN-based adaptive modulation and coding framework for OTFS underwater communication systems. The presented framework combines delay–Doppler feature extraction with model-agnostic meta-learning (MAML) to improve adaptation to rapidly varying underwater channel conditions. Experimental evaluations showed accuracies of 80–90% at high SNR and 63–76% at low SNR.

Li et al.^21^ developed a DL framework for underwater signal identification and classification in severe oceanic noise. The framework combines time–frequency representations with small-sample fine-tuning and depends on SSD-MobileNet-V2-FPNlite and EfficientNet architectures for detection and modulation recognition. Experiments showed 94.2% classification accuracy at 10 dB SNR and about 89% under low-SNR conditions.

Zhou et al.^22^ addressed UWA modulation classification by combining two complementary feature representations: high-resolution smoothed pseudo-Wigner-Ville distribution (SPWVD) time–frequency maps and higher-order statistical features that capture phase information. These features are processed with a dual-branch ResNet–Transformer architecture, in which residual connections ensure stable gradient flow and the transformer-based attention mechanism enables adaptive fusion of local (ResNet) and global (self-attention) contextual features. The presented approach achieved approximately 98.9% classification accuracy at high SNR and about 95.8% at low SNR, demonstrating robust performance across diverse channel conditions.

In addition, several three-dimensional hybrid modulation schemes have been proposed and investigated in the literature^23–27^, as summarized in Table 2. These studies primarily focus on hybrid modulation design, spectral-efficiency enhancement, constellation-space representation, frequency-diversity improvement, and error-rate performance rather than AMC. However, many of these techniques face practical challenges in UWA communication systems because they are highly sensitive to underwater channel distortions, Doppler effects, and hardware impairments. Consequently, AMC for three-dimensional hybrid modulation schemes in underwater acoustic environments remains largely unexplored. Frequency-indexed hybrid modulation offers a more practical alternative for UWA environments by jointly exploiting frequency indices and complex modulation symbols^28,29^. Within this context, FPK and FQAM are promising candidates for reliable underwater modulation classification. Representative frequency-indexed hybrid modulation techniques are summarized in Table 3.

**Table 2:** Summary of three-dimensional hybrid modulation techniques.

References	Year	Modulation technique	Signal dimensions	Key concept	Typical application
Başar et al.^23^	2013	OFDM, M-QAM / M-PSK, and Index Modulation	Subcarrier Index, Amplitude, Phase	Index modulation using active subcarriers	UWA and high-mobility channels
Xiao et al.^24^	2014	OFDM with Interleaved Subcarrier Index Modulation (OFDM-ISIM)	Subcarrier Index, Amplitude, Phase	Interleaved index modulation with diversity enhancement	Highly-correlated fading channels; high-mobility OFDM systems
Sun et al.^25^	2015	PQAM (PAM, QAM, and Polarization)	Amplitude, Phase, Polarization	Polarization-assisted hybrid modulation	Wireless and optical communications
Wen et al.^26^	2017	OFDM with Hybrid I/Q Index Modulation (OFDM-HIQ-IM)	Subcarrier Index, I-Dimension, Q-Dimension	Hybrid I/Q index modulation	Spectral-efficiency-oriented OFDM systems
De Gaudenzi et al.^27^	2019	APSK / Star-QAM	Ring Index, Amplitude, Phase	Multi-ring constellation representation	Satellite and broadband communications

**Table 3:** Frequency-indexed hybrid modulation schemes.

References	Year	Modulation technique	Signal dimensions	Key concept	Typical application
Wu et al.^28^	2016	FQAM	Frequency Index, Amplitude, Phase	Joint transmission of frequency index and QAM symbols	Interference-limited cellular systems and 5G cell-edge users
Kundu et al.^29^	2020	FPK	Frequency, Phase, Signal Correlation	Frequency-indexed phase modulation with correlation optimization	Power- and error-efficient digital modulation: AWGN and Rayleigh fading channels

## FPK and FQAM modulation

The FQAM has emerged as a promising modulation technique for mitigating interference by jointly exploiting frequency indexing and complex symbol modulation^30^. Performance analyses have shown that FQAM can outperform conventional QAM in terms of bit error rate (BER), frame error rate (FER), and signal-to-interference-plus-noise ratio (SINR), particularly in interference-limited conditions.

The FPK is a special case of FQAM in which only phase modulation is used. As a result, FPK produces constant-envelope signals that are inherently robust to nonlinear distortions. This concept has been examined in several prior studies^31^, in which expanded signal constellations were constructed by combining conventional orthogonal frequency-shift keying (FSK) with standard M-PSK using uniformly-spaced phase shifts.

In UWA environments, hybrid frequency-index modulation is particularly attractive given the severely limited available bandwidth, strong ambient noise, and frequent interference among multiple underwater nodes. Motivated by these characteristics, this work is concentrated on multiple modulation orders, including 2FPK, 8FPK, and 16FPK, as well as 4FQAM, 8FQAM, and 32FQAM, to comprehensively assess the proposed AMC framework capability across different spectral efficiencies and constellation complexities.

**Fig. 1:**
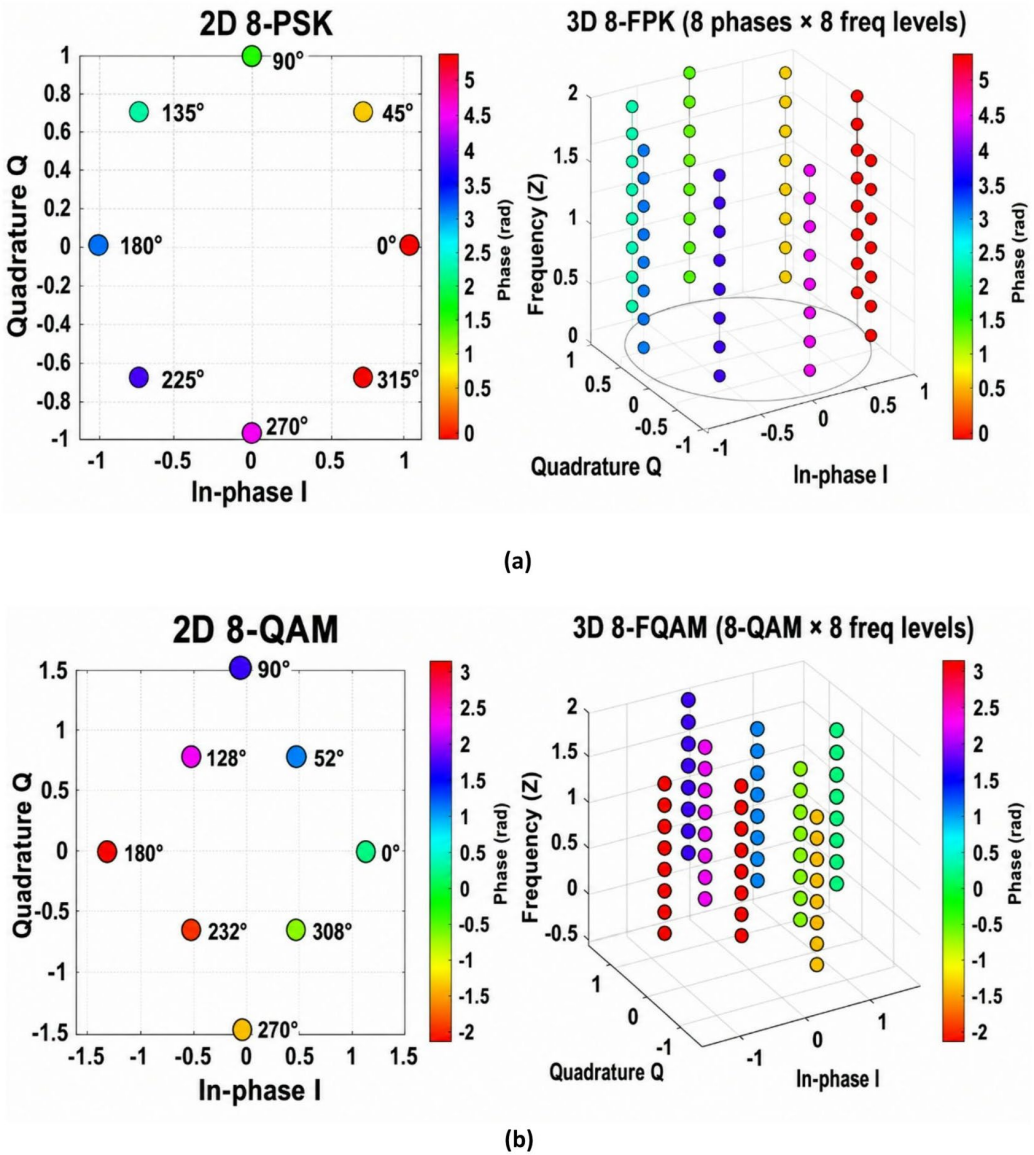
(**a**) 2D-PSK versus 3D-FPK constellations. (**b**) 2D-QAM versus 3D-FQAM constellations.

Figure 1 provides a comparative visualization of conventional two-dimensional (2D) modulation schemes and their corresponding three-dimensional (3D) frequency-index modulation counterparts. Specifically, the comparison covers 2D-PSK versus 3D-FPK and 2D-QAM versus 3D-FQAM. In conventional 2D-PSK constellations, information is conveyed solely through phase variations in the in-phase and quadrature (I–Q) plane. In contrast, 3D-FPK extends the signal representation by introducing an additional frequency dimension, with symbols distinguished jointly by phase modulation and frequency indexing. Similarly, conventional 2D-QAM encodes information using amplitude and phase variations, whereas 3D-FQAM further enhances the representation by integrating amplitude–phase modulation with frequency indexing.

### Theoretical analysis of the proposed three-dimensional constellation structure

To further explain the performance improvement achieved by the introduced frequency-domain constellation representation, a theoretical interpretation based on signal-space geometry and feature separability is provided. In conventional two-dimensional constellation mapping, modulation symbols are distributed only along the (I) and (Q) dimensions. Under severe underwater acoustic channel conditions, including multipath propagation, Doppler spread, and additive noise, adjacent constellation points may overlap significantly, reducing modulation separability and degrading classification performance.

In the proposed approach, an additional frequency-index dimension is incorporated into the signal representation, forming a three-dimensional signal space. This dimension increases the spatial separation between constellation points and improves the geometric discriminability of modulation patterns. Consequently, the resulting constellation structures are more robust to channel distortion and interference.

More specifically, the Euclidean distance between constellation points plays a critical role in determining modulation classification and detection performance. For two constellation points ${\mathrm{S}}_{i}\left({x}_{i},{y}_{i},{z}_{i}\right)$ and ${\mathrm{S}}_{j}\left({x}_{j},{y}_{j},{z}_{j}\right)$, the Euclidean distance in the proposed 3D signal space is expressed as:

1 $${d}_{ij}=\sqrt{{\left({x}_{i}-{x}_{j}\right)}^{2}+{\left({y}_{i}-{y}_{j}\right)}^{2}+{\left({z}_{i}-{z}_{j}\right)}^{2}}$$

Therefore, constellation points become less susceptible to overlap under severe underwater channel distortions. Compared with conventional 2D constellation representations, the additional frequency dimension increases the minimum distance between modulation symbols, thereby reducing symbol ambiguity and improving resistance to noise, multipath fading, and underwater channel impairments. This leads to enhanced inter-class separability and reduced intra-class overlap within the generated constellation images.

Furthermore, the proposed 3D constellation representation provides richer structural information for the employed CNN architectures, enabling more effective extraction of discriminative deep features. This advantage is particularly important for high-dimensional constellation configurations and low-SNR conditions, where conventional 2D constellation diagrams often suffer from severe cluster overlap and feature degradation. By distributing constellation points across a higher-dimensional feature space, the proposed method improves modulation distinguishability and contributes to the superior AMC performance observed across the experimental results.

## Experimental setup

### SC FPK/FQAM transmission system model

**Fig. 2:**
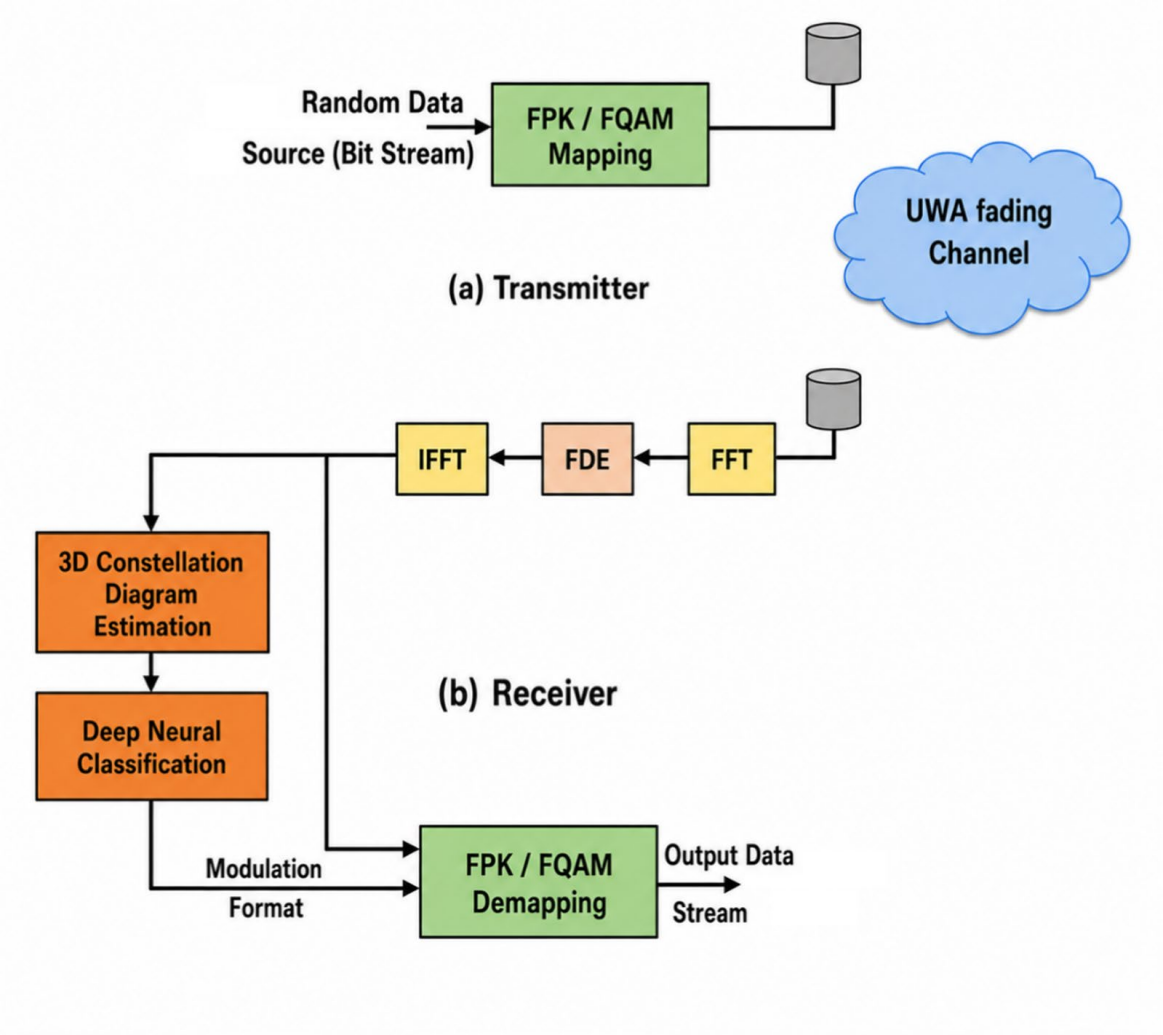
Proposed AMC for UWA-SC: (**a**) Transmitter, (**b**) Receiver.

This section presents the proposed AMC framework for the UWA-SC system, as illustrated in Fig. 2.

At the transmitter, a random data source generates a binary bit stream, which is mapped into FPK or FQAM symbols. The transmitted symbols are constructed from a full FQAM constellation by jointly combining discrete frequency indices with complex QAM symbols, forming a three-dimensional frequency–IQ representation. In this implementation, a reference amplitude of *A*=1 is used for normalization. FPK depends on a constant-envelope signal with information carried by frequency indices and phase states, whereas FQAM depends on variable-amplitude, unit-power-normalized QAM symbols (4–32 QAM) combined with frequency indexing. The number of frequency indices defining the third constellation dimension is ${M}_{f}\in \left\{\mathrm{2,8},\left.16\right\}\right.$ for FPK and ${M}_{f}\in \left\{\mathrm{4,8},\left.32\right\}\right.$ for FQAM.

The modulated signal propagates through a UWA channel, since acoustic waves propagate more efficiently in water than electromagnetic waves. The UWA channel is modelled using a measured impulse response obtained from the mobile acoustic communications experiment (MACE), specifically the second experiment^32^. The adopted MACE parameters were derived from real underwater measurements, and therefore they reflect realistic underwater acoustic propagation characteristics. To capture practical multipath effects typical for underwater environments, a six-tap channel realization is randomly selected from the MACE impulse response matrix. The channel vector is normalized to unit norm and mixed with the transmitted signal via linear convolution. AWGN is then introduced according to a specified target SNR.

**Fig. 3:**
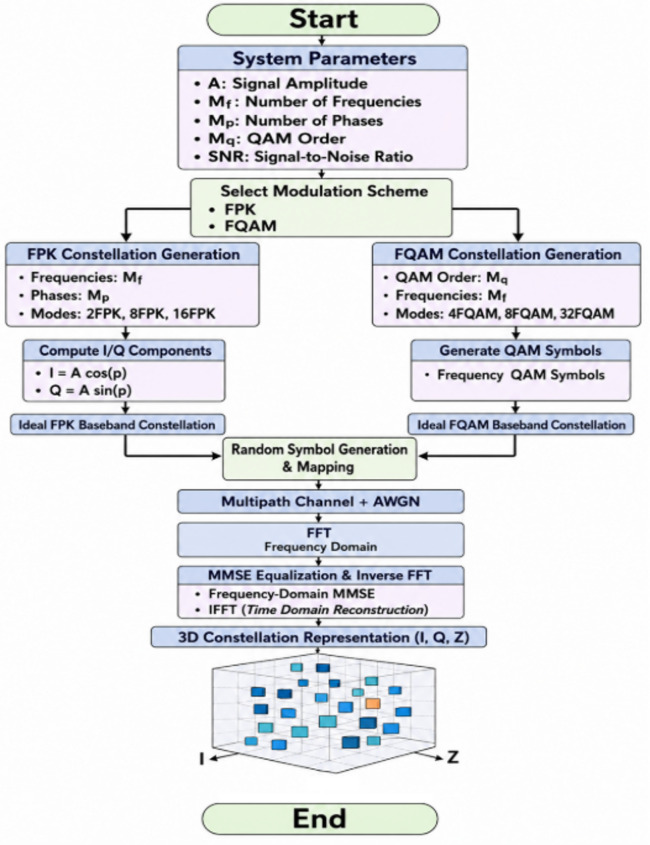
Flowchart of the SC 3D constellation generation process for FPK and FQAM modulation schemes. The framework supports multiple FPK and FQAM formats and depends on FFT-based LMMSE equalization before extracting the I, Q, and frequency components for 3D constellation visualization.

At the receiver, the signal is processed using a frequency-domain LMMSE equalizer to mitigate channel distortion. The equalizer is constructed using the FFT of the channel impulse response and a noise covariance matrix derived from the specified SNR. The received signal is transformed to the frequency domain, equalized, and then converted back to the time domain using an inverse FFT (IFFT), after which the signal is truncated to its original symbol length. From the equalized symbols, the I and Q components are extracted, while the frequency index associated with each transmitted symbol forms the third (*Z*) dimension of the constellation, resulting in a 3D constellation representation, as illustrated in Fig. 3.

The images are generated invisibly to support large-scale batch processing and are saved before being resized to a standardized resolution of 227 × 227 pixels, ensuring compatibility with CNN architectures.

For each SNR value in the range of − 10 dB to 30 dB (at 1 dB resolution), a fixed number of constellation images are generated for each modulation mode, resulting in a labelled dataset organized by SNR and modulation format. These 3D constellation images are fed into deep neural network classifiers. The trained models identify the transmitted modulation format, which is then used at the receiver to perform the appropriate FPK/FQAM demapping and recover the output data stream.

**Fig. 4:**
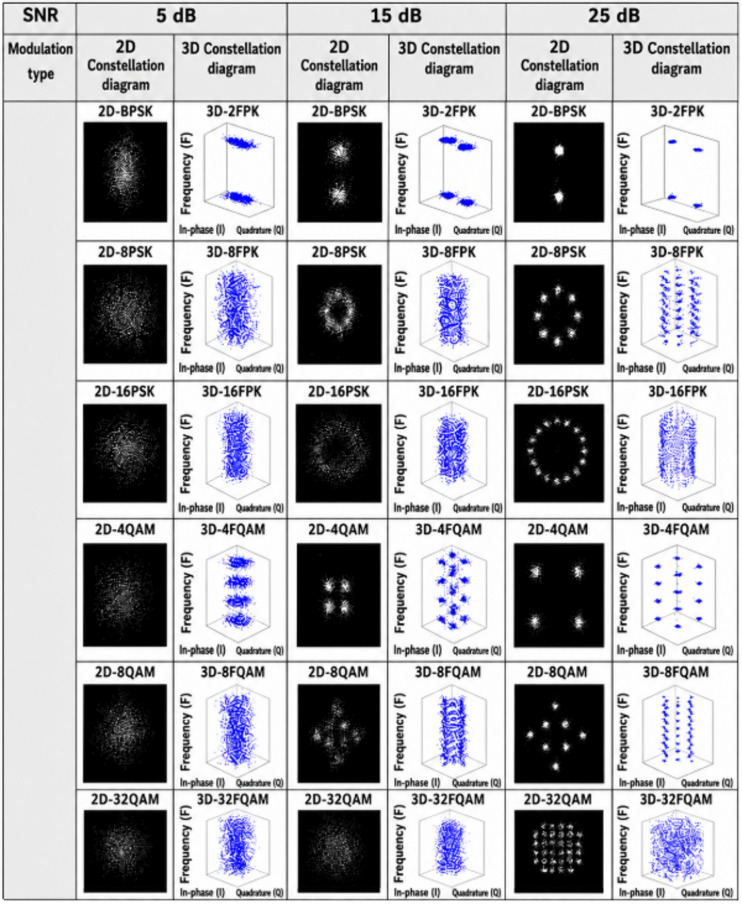
Conventional 2D and proposed 3D constellation diagrams for various modulation types in the UWA-SC system at different SNR levels (5 dB, 15 dB, and 25 dB).

Figure 4 shows that conventional 2D constellation diagrams degrade significantly at low SNR, increasing overlap between modulation schemes. In contrast, an additional frequency-index dimension reduces constellation overlap caused by multipath fading and additive noise. Unlike conventional 2D representations, the proposed structure preserves modulation-specific spatial patterns even at low SNR, facilitating more reliable CNN-based feature extraction across varying SNR levels. This added dimensionality provides more robust and discriminative features, supporting the effectiveness of 3D constellation images for deep learning–based modulation recognition.

### OFDM FPK/FQAM transmission system model

**Fig. 5:**
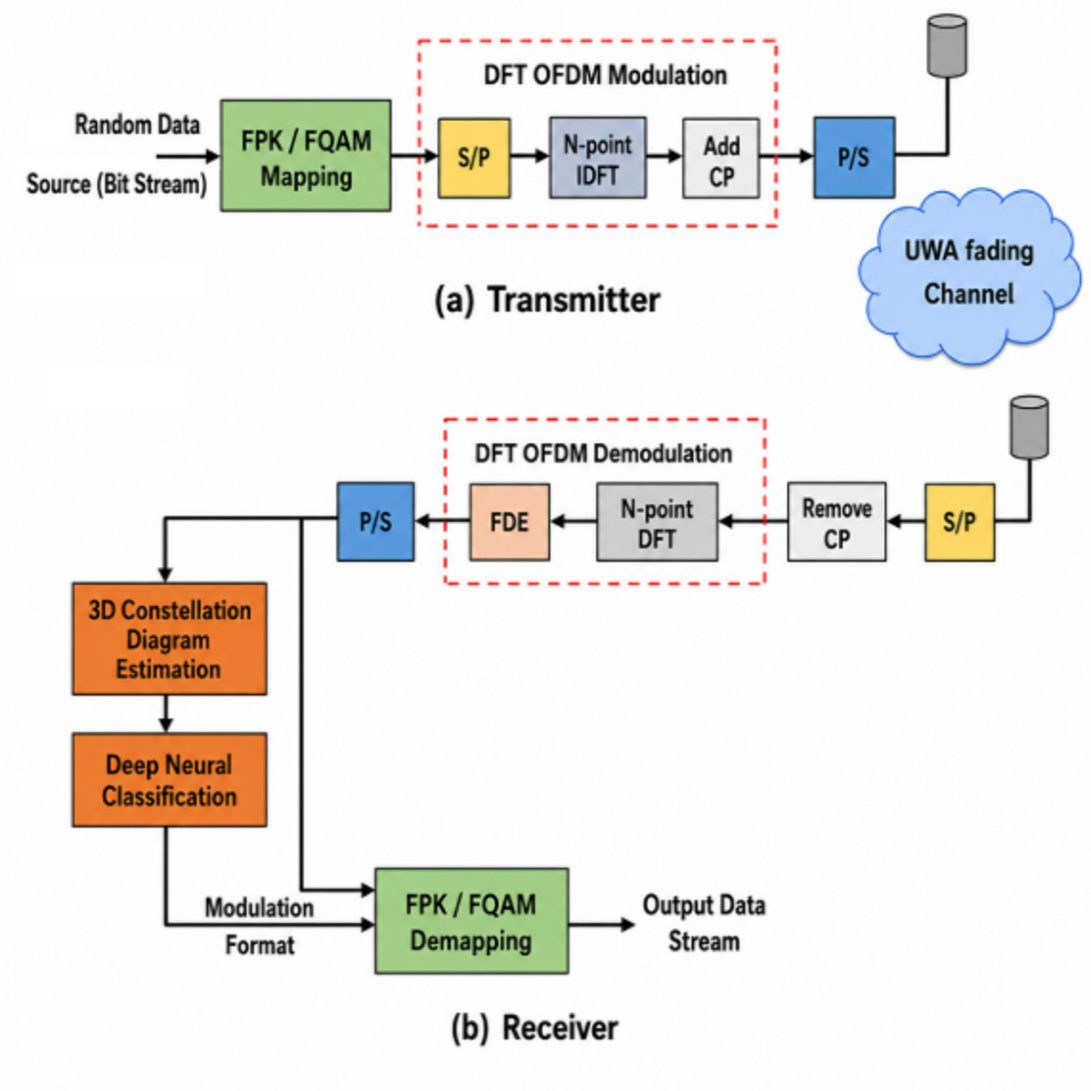
The Proposed AMC for UWA-OFDM: (**a**) Transmitter, (**b**) Receiver.

This section presents the proposed AMC framework for the UWA-OFDM system, as illustrated in Fig. 5.

A single-input single-output (SISO) OFDM system is considered for UWA communications, where a single transmitting hydrophone and a single receiving hydrophone are employed. The OFDM signal consists of *N* orthogonal subcarriers, each occupying a bandwidth of *Δ f*. Therefore, the total transmission bandwidth is expressed as:

2 *BW = N Δ f*

where *N* denotes the number of OFDM subcarriers, and *Δ f* represents the subcarrier spacing.

The simulation parameters adopted throughout this study were selected according to the MACE UWA channel configuration. Specifically, a carrier frequency of 15 kHz and a transmission bandwidth of 10 kHz were used. The OFDM transmission scheme has 1000 subcarriers and a cyclic prefix (CP) of length 16 samples.

At the transmitter, a random binary data stream is mapped into complex-valued symbols using FPK and FQAM modulation schemes, including 2FPK, 8FPK, 16FPK, 4FQAM, 8FQAM, and 32FQAM constellations. A reference signal amplitude of *A* = 1 is adopted for normalization. In the FPK scheme, information is conveyed through frequency states and phase states, while maintaining a constant-envelope signal. In contrast, FQAM depends on unit-power normalized QAM symbols combined with frequency indexing. The number of frequency states defining the third constellation dimension is given by ${M}_{f}\in \left\{\mathrm{2,8},\left.16\right\}\right.$ for FPK and ${M}_{f}\in \left\{\mathrm{4,8},\left.32\right\}\right.$ for FQAM.

$${\mathrm{L}\mathrm{e}\mathrm{t}}\quad\,{\mathbf{X}} = \left[ {X_{0} ,X_{1} , \ldots ,X_{N - 1} } \right]^{T} \in {\mathbb{C}}^{N \times 1}$$

denote the transmitted frequency-domain symbol vector.

As illustrated in Fig. 5, a serial-to-parallel (S/P) conversion is first applied to distribute the mapped symbols across the OFDM subcarriers. Subsequently, OFDM modulation is performed using an *N*-point IDFT. The resulting time-domain OFDM signal is expressed as:

3 $${\mathbf{X}}_{{\it{I}}{\it{D}}{\it{F}}{\it{T}}}={\mathbf{F}}_{N}^{-1}\mathbf{X}$$

where ${\mathbf{F}}_{N}^{-1}$∈ ${\mathbb{C}}^{N\times N}$ is an *N* × *N* complex-valued IDFT matrix, and ${\mathbf{X}}_{{\it{I}}{\it{D}}{\it{F}}{\it{T}}}$ ∈ ${\mathbb{C}}^{N\times 1}$ represents the time-domain OFDM signal before CP insertion. To mitigate inter-symbol interference (ISI) caused by multipath propagation in the UWA channel, a CP of length ${L}_{\mathrm{C}\mathrm{P}}$ ​ is appended to each OFDM symbol. The transmitted OFDM signal with CP is therefore given by:

4 $$\widetilde{\mathbf{X}}=\mathbf{P}{\mathbf{X}}_{{\it{I}}{\it{D}}{\it{F}}{\it{T}}}$$

where $\mathbf{P}$ denotes the CP insertion matrix, $\widetilde{\mathbf{X}}$ represents the transmitted OFDM signal after CP insertion. At the receiver, the CP is removed before applying the *N*-point DFT to recover the received signal in the frequency domain. Assuming perfect synchronization and ideal CP removal, the received frequency-domain signal can be expressed as:

5 $$\mathbf{Y}={{\boldsymbol{\upeta}}}_{\it{D}\it{F}\it{T}}\mathbf{H}\mathbf{X}+\mathbf{N}$$

where $\mathbf{Y}$ ∈ ${\mathbb{C}}^{N\times 1}$ is the received frequency-domain signal vector, $\mathbf{H}$ ∈ ${\mathbb{C}}^{N\times N}$ represents the channel frequency–response matrix of the UWA channel, $\mathbf{N}$ ∈ ℂ^*N*×1^ denotes the additive white Gaussian noise (AWGN) vector, and ${{\boldsymbol{\upeta}}}_{\it{D}\it{F}\it{T}}$ ∈ ${\mathbb{C}}^{N\times N }$ denotes the DFT interference matrix accounting for inter-carrier interference effects.

For compact representation, the equivalent composite channel matrix is defined as:

6 $${\boldsymbol{\Pi}}={{\boldsymbol{\upeta}}}_{\it{D}\it{F}\it{T}}\mathbf{H}$$

Accordingly, the received signal model can be rewritten as:

7 $$\mathbf{Y}={\boldsymbol{\Pi}}\mathbf{X}+\mathbf{N}$$

Frequency-domain equalization is subsequently applied using the LMMSE equalizer. The estimated transmitted symbol vector is obtained as:

8 $$\widehat{\mathbf{X}}={\boldsymbol{\Psi}}.\mathbf{Y}$$

where ${\boldsymbol{\Psi}}$ ∈ ${\mathbb{C}}^{N\times N}$ denotes the LMMSE equalization matrix. The LMMSE equalizer is expressed as:

9 $${\boldsymbol{\Psi}}={\left({{\boldsymbol{\Pi}}}^{H}{\boldsymbol{\Pi}}+\frac{1}{SNR}{\mathbf{I}}_{{\it{N}}}\right)}^{-1}{{\boldsymbol{\Pi}}}^{H}$$

${\mathrm{w}\mathrm{h}\mathrm{e}\mathrm{r}\mathrm{e}\;{\boldsymbol{\Pi}}}^{H}$ denotes the Hermitian transpose of the equivalent channel matrix, ${\mathbf{I}}_{{\it{N}}}$ ∈ ${\mathbb{C}}^{N\times N}$ is the *N* × *N* identity matrix. Substituting the received signal model into the equalization expression yields:

10 $$\widehat{\mathbf{X}}={\boldsymbol{\Psi}}{\boldsymbol{\Pi}}\mathbf{X}+{\boldsymbol{\Psi}}\mathbf{N}$$

Assuming accurate channel equalization, we get:

11 $$\boldsymbol{\Psi} \boldsymbol{\Pi} \approx \mathbf{ I}_{{\it{N}}}$$

The estimated transmitted symbols can be approximated as:

12 $$\widehat{\mathbf{X}}=\mathbf{X}+{{\boldsymbol{\Phi}}}_{F}\quad\mathrm{a}\mathrm{n}\mathrm{d}\quad {{\boldsymbol{\Phi}}}_{F}={\boldsymbol{\Psi}}\mathbf{N}$$

${{\boldsymbol{\Phi}}}_{F}$ denotes the equivalent post-equalization noise vector.

**Fig. 6:**
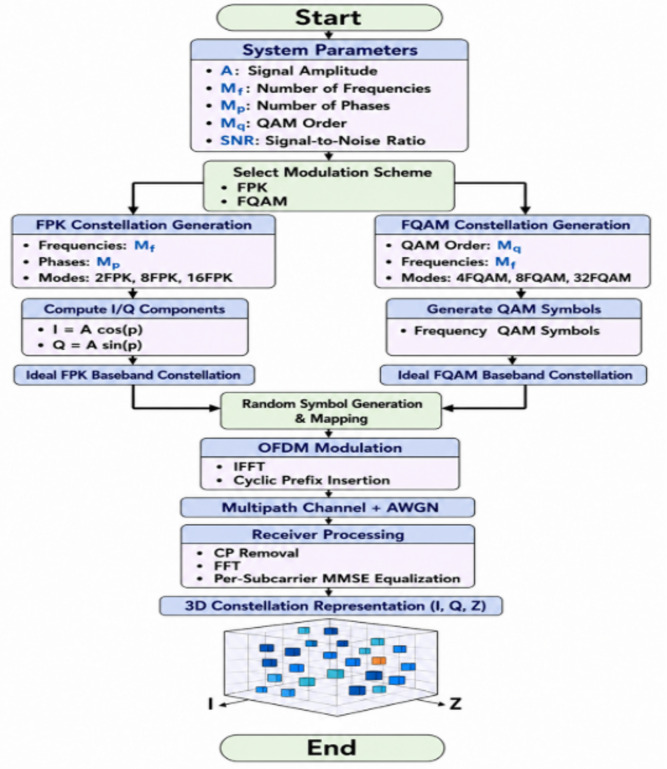
Flowchart of the OFDM-based 3D constellation generation process for FPK and FQAM modulation schemes, employing CP insertion and per-subcarrier LMMSE equalization before extracting the in-phase (I), quadrature (Q), and frequency (Z) components.

After equalization, the recovered frequency-domain symbols are converted back into a serial data stream and utilized to generate 3D constellation scatter representations. In the resulting visualization, the (I) and (Q) components form the horizontal axes, while the associated frequency-state information is mapped onto the third axis (f), as illustrated in Fig. 6.

These 3D constellation images are directly fed into DL-based AMC classifiers, namely AlexNet and VGG-19, to automatically identify the transmitted modulation format among 2FPK, 8FPK, 16FPK, 4FQAM, 8FQAM, and 32FQAM. Once the modulation scheme is recognized, the corresponding FPK/FQAM demapping process is applied to recover the output binary data stream.

**Fig. 7:**
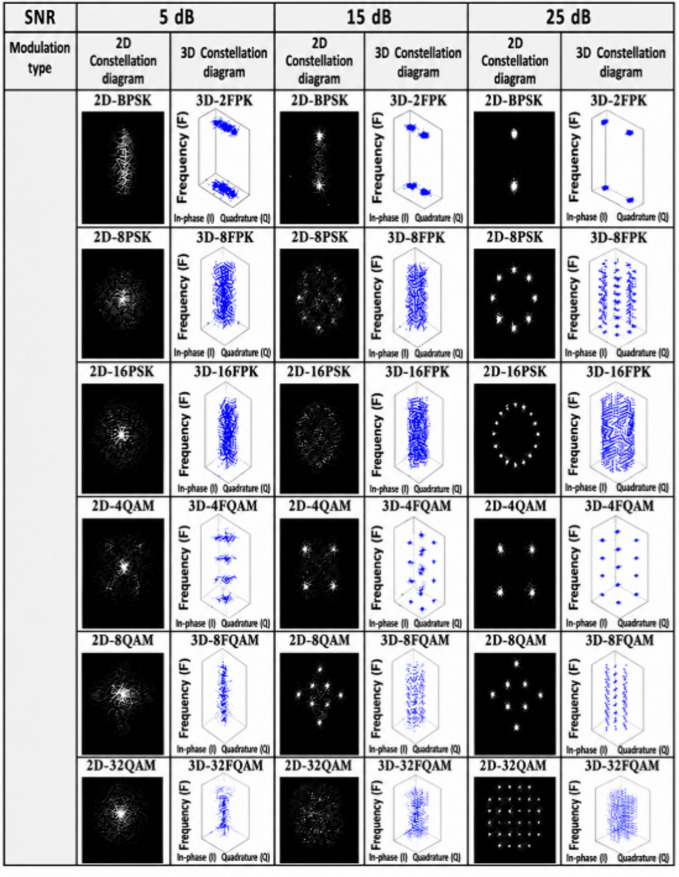
Constellation diagrams for various modulation types in the proposed UWA-OFDM system at SNRs of 5, 15 and 25 dB, showing a comparison between conventional 2D I–Q constellations and the corresponding 3D frequency–IQ representations.

Figure 7 illustrates 2D and 3D constellation diagrams for an OFDM system under three SNR conditions: 5 dB, 15 dB, and 25 dB. Results are shown for multiple modulation schemes, including FPK and FQAM. As the SNR decreases, conventional 2D constellations experience severe cluster spreading and symbol overlap due to underwater channel distortion. In contrast, the proposed 3D frequency-IQ representation maintains clearer geometric separation between modulation classes by distributing signal points across an additional frequency dimension.

### Dataset description

In this experiment, two distinct datasets were constructed to evaluate the performance of DL-based AMC over a UWA channel modelled using the MACE channel model. The datasets correspond to SC transmission with LMMSE equalization and OFDM-DFT transmission with LMMSE equalization. All simulations and dataset generation were implemented using MATLAB R2020b, where the MACE channel model was employed to emulate realistic UWA propagation conditions.

For both SC and OFDM transmission systems, six modulation schemes were considered, namely 2FPK, 4FQAM, 8FPK, 8FQAM, 16FPK, and 32FQAM, and were applied consistently across all experiments. Each modulation scheme was evaluated over 41 SNR levels ranging from − 10 to 30 dB in 1 dB increments. At each modulation–SNR combination, 125 independent constellation diagram images were generated to capture channel-induced variability. The selected number of samples provided a balance between dataset diversity and computational complexity, while ensuring stable CNN training and unbiased performance evaluation across all investigated modulation formats and SNR conditions. In addition, variability was further enhanced through random channel realizations, AWGN corruption, and different equalized constellation structures generated under the MACE underwater acoustic channel model. AWGN was added after convolution with the MACE channel impulse response to achieve the desired SNR levels.

As a result, each dataset (SC and OFDM) contains approximately 31,000 constellation diagram images, yielding a total of 62,000 images across both transmission schemes. The generated constellation diagrams were randomly shuffled and divided into 70% for training, 20% for testing, and 10% for validation, ensuring an unbiased evaluation of the learning models. These datasets were used to train and evaluate the AlexNet, VGG-19, and ResNet50 DL models for modulation classification under varying SNR conditions, enabling a comprehensive assessment of AMC performance in realistic MACE-based UWA channel environments.

#### Pre-trained AlexNet model

In this work, the pre-trained AlexNet model is employed as one of the DL architectures for automatic modulation recognition^33^. AlexNet consists of five convolutional layers with 96, 256, 384, 384, and 256 filters, respectively. Each convolutional layer has a 3 × 3 kernel. While most convolutional layers adopt a stride of 1, the first convolutional layer is configured with a stride of 4. The corresponding padding values for the five layers are 0, 2, 1, 1, and 1, respectively.

A ReLU activation function follows each convolutional layer, and 3 × 3 max-pooling is applied to extract the most discriminative features and reduce spatial dimensionality. To adapt AlexNet to the modulation classification task, the original classification layer was replaced with a new fully-connected layer consisting of six neurons, corresponding to the six modulation classes considered in this work. The softmax activation function is used in the output layer for final classification. In addition, the validation frequency during training was increased from 3 to 100 iterations to improve performance monitoring. The detailed architectural parameters of the AlexNet model are summarized in Table 4. The modified AlexNet architecture is used for training and testing under both SC and OFDM transmission schemes.

**Table 4:** Statistical parameters for training.

Parameter	AlexNet	VGG-19	ResNet50
Training Time (Min: Sec)	13:32	39:16	66:44
Epochs	6	6	6
Total Iterations	15,150	15,150	15,150
Iterations/Epoch	2525	2525	2525
Validation Frequency (Iterations)	100	100	100
Learning Rate	0.0001	0.0001	0.0001
Optimizer	SGDM	SGDM	SGDM
Learning Rate Schedule	Fixed	Fixed	Fixed
Mini-Batch Size	10	10	10
Loss Function	Cross-Entropy	Cross-Entropy	Cross-Entropy
Initialization	ImageNet pre-trained	ImageNet pre-trained	ImageNet pre-trained
Data Augmentation	Reflection and Translation	Reflection and Translation	Reflection and Translation
Hardware Resources	Single GPU	Single GPU	Single GPU

#### Pre-trained VGG-19 model

The pre-trained VGG-19 network is also utilized to evaluate the performance of DL–based AMC in UWA systems^34^. The VGG-19 architecture comprises 16 convolutional layers, where the number of filters is arranged as 64, 64, 128, 128, 256, 256, 256, 256, and 512 in the last eight layers, respectively. All convolutional layers have a 3 × 3 kernel, a stride of 1, and padding of 1, and each is followed by a ReLU activation function. Feature reduction is performed using 2 × 2 max-pooling layers, which enable effective extraction of spatial features. To tailor the pre-trained VGG-19 model to the modulation recognition task, the input image size was adjusted to 227 × 227 × 3, and the original fully-connected classification layer was replaced with a new layer corresponding to the required number of six modulation classes. The softmax activation function is applied at the output layer to generate class probabilities. The statistical training parameters of the VGG-19 model are provided in Table 4. The modified VGG-19 architecture is used for training and testing under both SC and OFDM transmission schemes.

#### Pre-trained ResNet50 model

The proposed model is based on the pre-trained ResNet50 architecture^35^, consisting of 53 convolutional layers with gradually increasing filter depths. Specifically, the architecture includes 7 layers with 64 filters, 8 layers with 128 filters, 16 layers with 256 filters, 11 layers with 512 filters, 7 layers with 1024 filters, and 4 layers with 2048 filters. All convolutional layers employ a 3 × 3 kernel, a stride of 1, and zero padding, except the initial convolutional layer, which has a 3 × 3 kernel, a stride of 2, and a padding of 3. ReLU activation functions are applied after each convolutional layer. To enhance feature selection, 3 × 3 Max pooling is integrated. The network ends with a fully-connected layer that has 6 neurons, with each neuron representing one of the six modulation classes, and it has a Softmax activation for the final classification. By the training strategy, the validation frequency was increased from 3 to 100 to improve model generalization. The detailed architectural parameters of the ResNet50 model are summarized in Table 4.

## Simulation results and comparative analysis

### Results for the proposed SC FPK/FQAM and OFDM FPK/FQAM systems

In this paper, we evaluate the performance of DL–based AMC for UWA communication systems using pre-trained AlexNet and VGG-19 models. The study focuses exclusively on FPK and FQAM modulation schemes, namely 2FPK, 4FQAM, 8FPK, 8FQAM, 16FPK, and 32FQAM, employing 3D constellation scatter representations that incorporate frequency-domain information. Two transmission schemes are considered: SC transmission with LMMSE equalization and OFDM-DFT transmission with LMMSE equalization.

The performance of the proposed AMC framework is evaluated over a wide SNR range from − 10 dB to 30 dB, with detailed results presented in the form of classification accuracy curves and comparative tables.

**Fig. 8:**
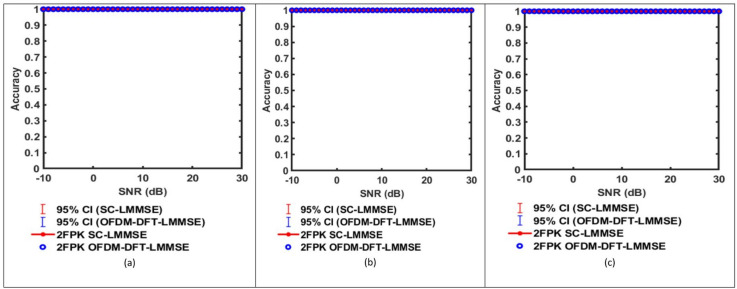
Classification accuracy with 95% confidence intervals for 2FPK modulation over the UWA channel under SC-LMMSE and OFDM-DFT-LMMSE transmission schemes using (**a**) AlexNet, (**b**) VGG-19, and (**c**) ResNet50. Error bars indicate 95% confidence intervals, and curves are smoothed for visual clarity.

For 2FPK modulation, both SC-LMMSE and OFDM-DFT-LMMSE schemes achieve a nearly-perfect classification accuracy approaching 100% across the entire SNR range for AlexNet, VGG-19, and ResNet50 models, as illustrated in Fig. 8. This behaviour is attributed to the low modulation order and the high separability of the constellation points, which make the modulation highly robust against noise, multipath effects, and underwater channel distortions. Consequently, only negligible performance differences are observed between the SC and OFDM transmission frameworks for this modulation format, as all evaluated CNN architectures maintain consistently-stable classification performance over the investigated SNR range.

**Fig. 9:**
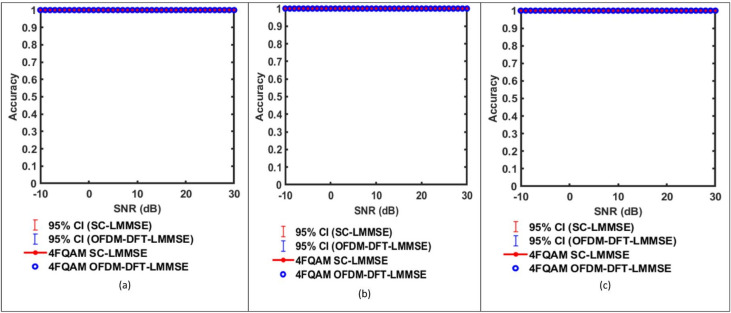
Classification accuracy with 95% confidence intervals for 4FQAM modulation over the UWA channel under SC-LMMSE and OFDM-DFT-LMMSE transmission schemes using (**a**) AlexNet, (**b**) VGG-19, and (**c**) ResNet50. Error bars indicate 95% confidence intervals, and curves are smoothed for visual clarity.

For 4FQAM modulation, both SC-LMMSE and OFDM-DFT-LMMSE schemes achieve classification accuracies approaching 100% across the entire investigated SNR range for AlexNet, VGG-19, and ResNet50 models, as illustrated in Fig. 9. The obtained results demonstrate highly-stable and reliable modulation recognition performance for all evaluated CNN architectures, confirming that low-order QAM modulation formats exhibit strong separability even under challenging UWA channel conditions. Similar to the 2FPK case, only negligible performance differences are observed between the SC and OFDM transmission frameworks, indicating that both schemes provide highly-robust classification capability for low-order modulation formats.

**Fig. 10:**
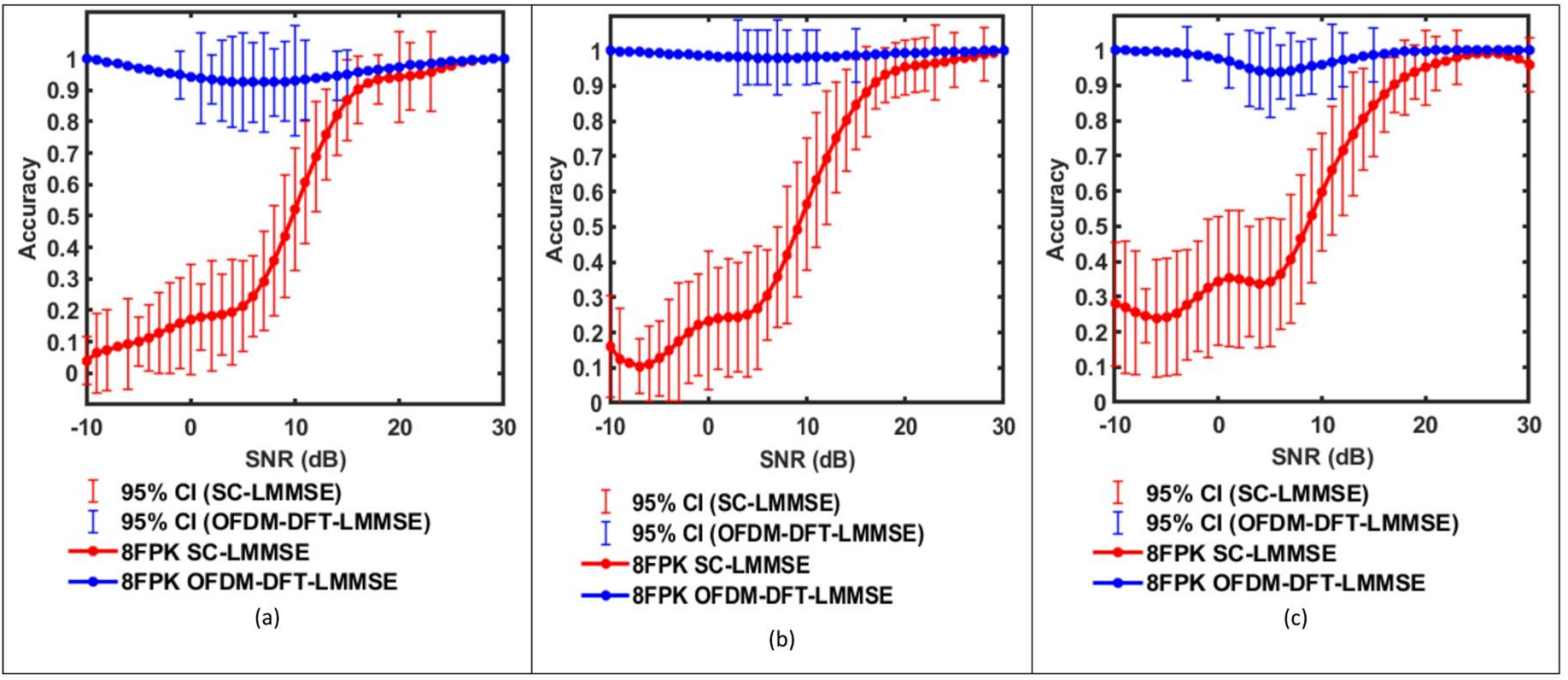
Classification accuracy with 95% confidence intervals for 8FPK modulation over the UWA channel under SC-LMMSE and OFDM-DFT-LMMSE transmission schemes using (**a**) AlexNet, (**b**) VGG-19, and (**c**) ResNet50. Error bars indicate 95% confidence intervals, and curves are smoothed for visual clarity.

For 8FPK modulation, a clear performance difference is observed between the SC-LMMSE and OFDM-DFT-LMMSE transmission schemes, as illustrated in Fig. 10. Under the SC-LMMSE framework, AlexNet, VGG-19, and ResNet50 exhibit relatively low classification accuracy at low SNR values, followed by a gradual improvement as the SNR increases. For example, the classification accuracy rises from approximately 10–25% at low SNR levels to nearly 100% at high SNR values. In contrast, the OFDM-DFT-LMMSE scheme maintains consistently-high classification accuracy exceeding 95% across the entire SNR range for all evaluated CNN architectures. The superior performance of the OFDM framework is mainly attributed to its ability to reduce frequency-selective fading and preserve modulation-specific constellation characteristics under noisy UWA conditions.

**Fig. 11:**
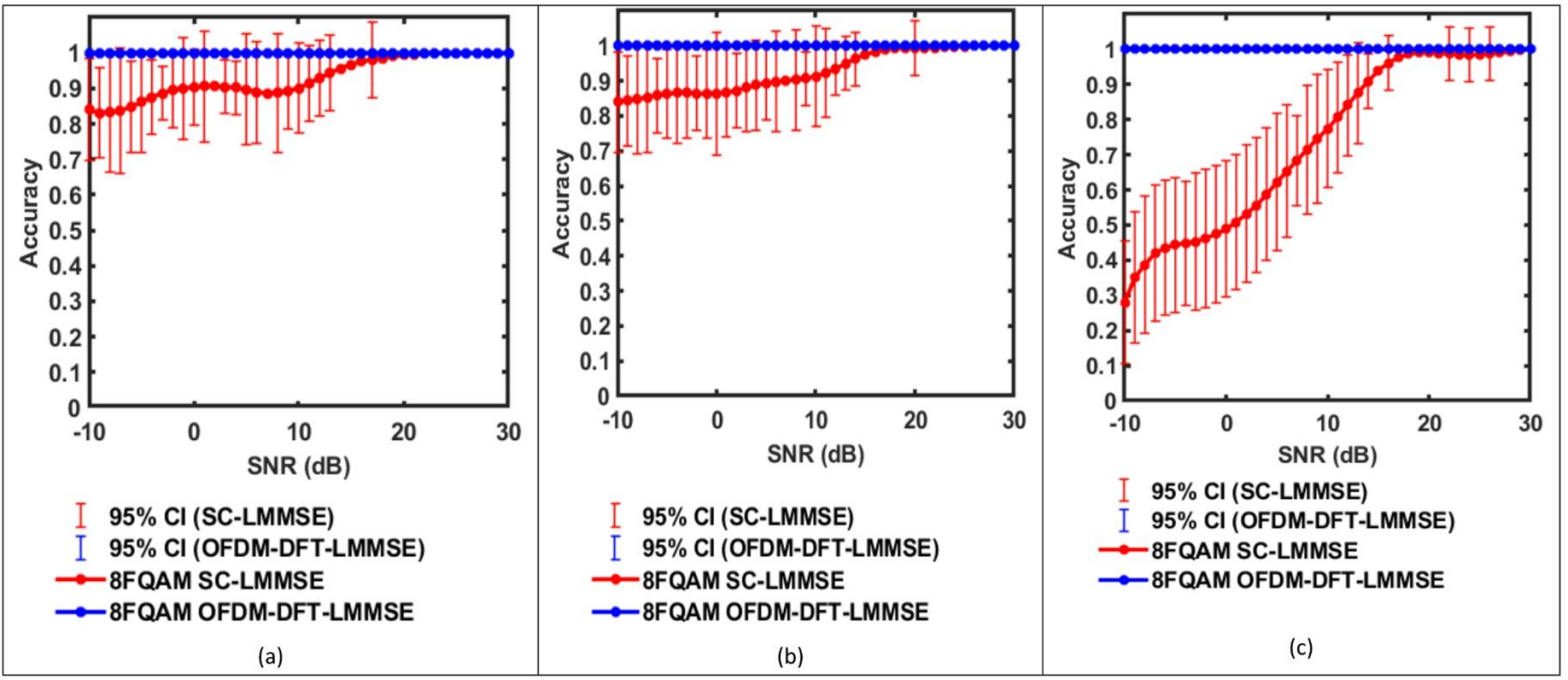
Classification accuracy with 95% confidence intervals for 8FQAM modulation over the UWA channel under SC-LMMSE and OFDM-DFT-LMMSE transmission schemes using (**a**) AlexNet, (**b**) VGG-19, and (**c**) ResNet50. Error bars indicate 95% confidence intervals, and curves are smoothed for visual clarity.

For 8FQAM modulation, the SC-LMMSE transmission scheme exhibits noticeable performance degradation and fluctuations in the low- and medium-SNR regions for AlexNet, VGG-19, and ResNet50 models, as illustrated in Fig. 11. At low SNR levels, the classification accuracy ranges approximately from 45 to 90%, depending on the employed CNN architecture, followed by gradual improvement as the SNR increases. This behaviour reflects the increased sensitivity of higher-order FQAM modulation to noise, multipath propagation, and underwater channel distortions. In contrast, the OFDM-DFT-LMMSE scheme maintains nearly perfect classification accuracy close to 100% across the entire SNR range for all evaluated CNN models, enhancing classification stability under severe UWA channel impairments. Furthermore, VGG-19 and ResNet50 generally provide slightly better low-SNR performance compared with AlexNet due to their deeper feature extraction capabilities.

**Fig. 12:**
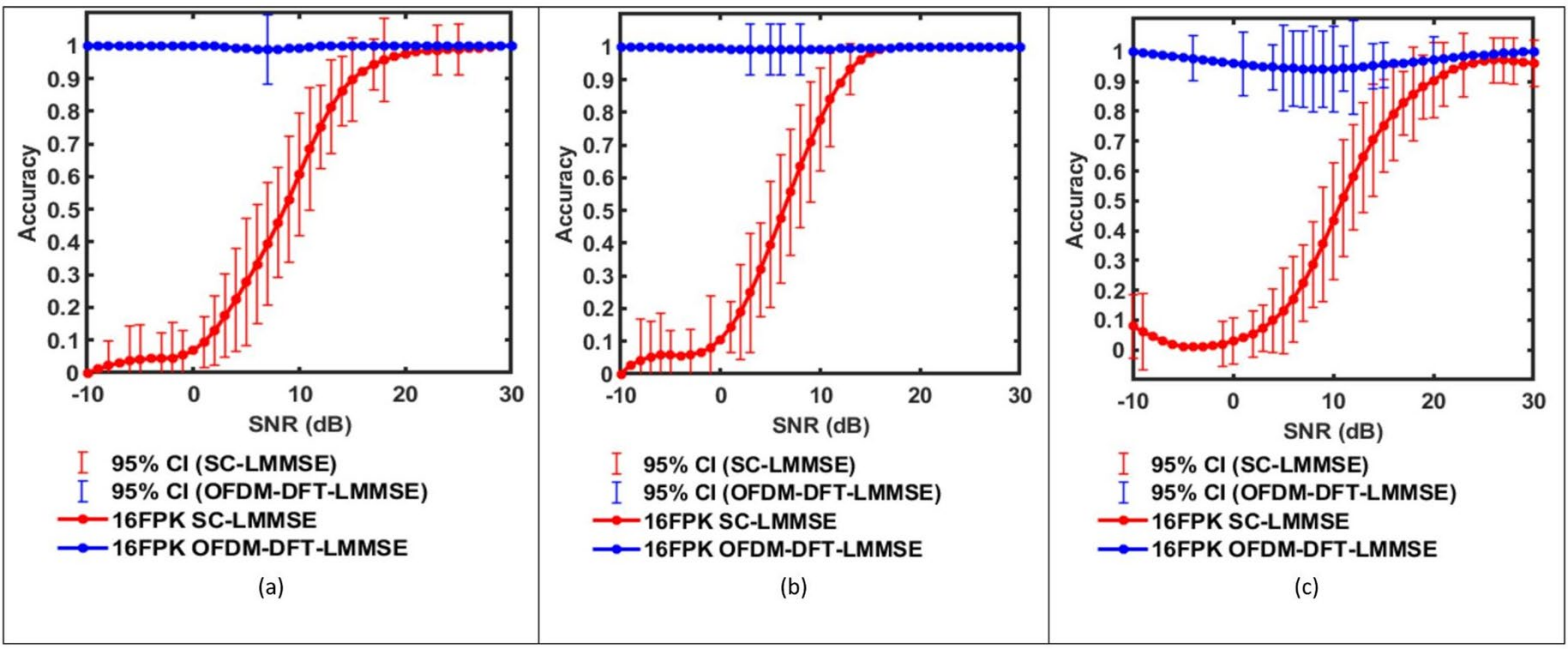
Classification accuracy with 95% confidence intervals for 16FPK modulation over the UWA channel under SC-LMMSE and OFDM-DFT-LMMSE transmission schemes using (**a**) AlexNet, (**b**) VGG-19, and (**c**) ResNet50. Error bars indicate 95% confidence intervals, and curves are smoothed for visual clarity.

For 16FPK modulation, the performance gap between the SC-LMMSE and OFDM-DFT-LMMSE transmission schemes becomes significantly more pronounced, as illustrated in Fig. 12. Under the SC-LMMSE framework, AlexNet, VGG-19, and ResNet50 exhibit severe classification accuracy degradation at low SNR values, where the accuracy approaches nearly 0% for some models. The classification performance then gradually improves with the increase in SNR, reaching near-perfect accuracy only at high SNR levels. In contrast, the OFDM-DFT-LMMSE scheme maintains consistently high classification accuracy close to 100% across the entire investigated SNR range for all evaluated CNN architectures. The significant improvement achieved by OFDM-DFT-LMMSE reveals that frequency-domain equalization effectively preserves the structural characteristics of high-order FPK constellations, even under severe underwater channel distortion.

**Fig. 13:**
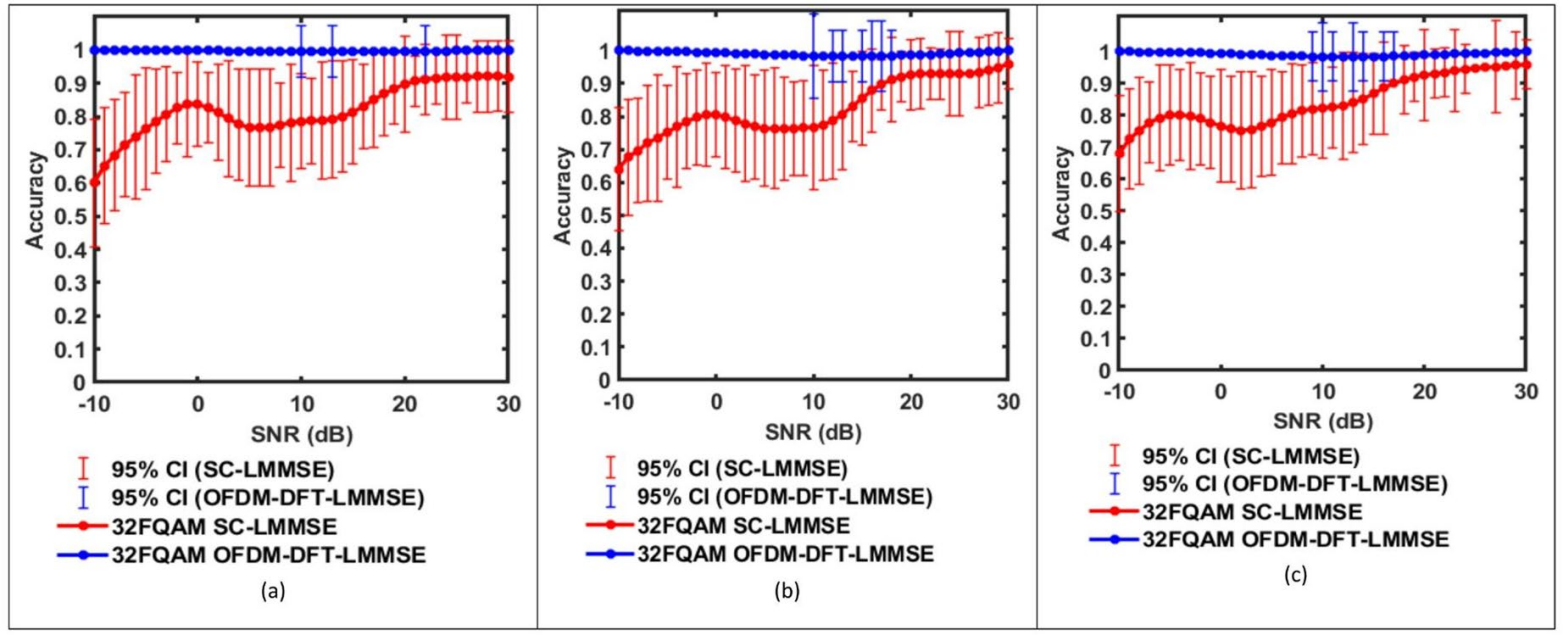
Classification accuracy with 95% confidence intervals for 32FQAM modulation over the UWA channel under SC-LMMSE and OFDM-DFT-LMMSE transmission schemes using (**a**) AlexNet, (**b**) VGG-19, and (**c**) ResNet50. Error bars indicate 95% confidence intervals, and curves are smoothed for visual clarity.

For 32FQAM modulation, which represents one of the most challenging high-order modulation scenarios, the SC-LMMSE transmission scheme exhibits the lowest classification accuracy and the strongest dependence on SNR, particularly in the low- and medium-SNR regions, as illustrated in Fig. 13. Under the SC-LMMSE framework, AlexNet, VGG-19, and ResNet50 achieve classification accuracies ranging approximately from 75 to 95%, with noticeable fluctuations caused by the increased sensitivity of high-order modulation formats to noise, multipath propagation, and underwater channel distortions. Although the classification accuracy gradually improves as the SNR increases, the obtained performance remains inferior to that achieved using the OFDM-DFT-LMMSE scheme.

In contrast, the proposed OFDM-DFT-LMMSE framework maintains consistently high classification accuracy close to 98–100% across the entire investigated SNR range for AlexNet, VGG-19, and ResNet50 models. The stable performance achieved by the OFDM-based framework indicates that the combination of OFDM processing and 3D constellation mapping effectively reduces constellation distortion and improves modulation distinguishability for dense constellation configurations. These results further demonstrate the effectiveness of the OFDM-based framework in preserving discriminative modulation features and enabling reliable AMC for high-order FQAM signals in multipath-dominated UWA environments.

Overall, the obtained results demonstrate that the OFDM-DFT-LMMSE approach consistently outperforms the SC-LMMSE approach, particularly for dense constellation modulation schemes and at reduced signal quality levels. The observed performance improvements become more significant as the modulation order increases, confirming that OFDM processing provides more discriminative and robust constellation representations for AMC.

In addition, the improved low-SNR performance achieved by VGG-19 and ResNet50 is likely related to their deeper hierarchical feature extraction capabilities, which enable more effective learning of subtle constellation-space variations compared with the shallower AlexNet architecture. They achieve slightly better classification performance than that of AlexNet, especially under challenging SNR conditions. Collectively, these findings validate the effectiveness of integrating OFDM signal processing with DL-based AMC frameworks for achieving reliable and robust modulation classification in severe UWA channel environments.

### Comparative analysis

Tables 5, 6 and 7 present a comparative evaluation of AlexNet, VGG-19, and ResNet50 models using the same dataset and identical UWA-MACE channel parameters to separately investigate the effects of constellation dimensionality and transmission schemes on modulation classification performance. The presented comparisons were carefully organized to isolate the contributions of 2D versus 3D constellation representations, as well as SC-LMMSE and OFDM-DFT-LMMSE processing frameworks.

**Table 5:** Comparison of AlexNet, VGG-19, and ResNet50 Models Using 2D and 3D Constellation Diagrams at 0 dB SNR.

Modulation type	BPSK	4QAM	8PSK	8QAM	16PSK	32QAM
AlexNet SC-LMMSE (2D Constellation Diagrams),^36^(2025)	**1**	**0**	**0.04**	**0.76**	**0**	**0.56**
VGG-19 SC- LMMSE (2D Constellation Diagrams),^37^(2025)	**1**	**0**	**0**	**0.32**	**0.08**	**0**
ResNet50 SC- LMMSE (2D Constellation Diagrams),^36^(2025)	**1**	**0.2**	**0.04**	**0.2**	**0**	**0.68**
AlexNet OFDM-DFT, LMMSE (2D Constellation Diagrams)	1	0.92	0.1	0.99	0.74	0.59
VGG-19 OFDM-DFT, LMMSE (2D Constellation Diagrams)	1	0.95	0.5	0.94	0.08	0.44
ResNet50 OFDM-DFT, LMMSE (2D Constellation Diagrams)	1	0.9	0.42	0.98	0.04	0.84

Modulation Type	2-FPK	4-FQAM	8-FPK	8-FQAM	16-FPK	32-FQAM
AlexNet SC-LMMSE (3D Constellation Diagrams)	**1**	**1**	**0.17**	**0.9**	**0.07**	**0.84**
VGG-19 SC- LMMSE (3D Constellation Diagrams)	**1**	**1**	**0.23**	**0.86**	**0.11**	**0.8**
ResNet50 SC- LMMSE (3D Constellation Diagrams)	**1**	**1**	**0.98**	**0.49**	**0.03**	**0.77**
AlexNet OFDM-DFT, LMMSE (3D Constellation Diagrams)	1	1	0.94	1	1	1
VGG-19 OFDM-DFT, LMMSE (3D Constellation Diagrams)	1	1	0.99	1	0.99	0.8
ResNet50 OFDM-DFT, LMMSE (3D Constellation Diagrams)	1	1	0.98	1	0.96	0.99

**Table 6:** Comparison of AlexNet, VGG-19, and ResNet50 models using 2D and 3D constellation diagrams at 10 dB SNR.

Modulation Type	BPSK	4QAM	8PSK	8QAM	16PSK	32QAM
AlexNet SC-LMMSE (2D Constellation Diagrams)^36^ (2025)	1	0.36	0.4	1	0.12	0.88
VGG-19 SC- LMMSE (2D Constellation Diagrams)^37^ (2025)	1	0.6	0.04	1	0.72	0.28
ResNet50 SC-LMMSE (2D Constellation Diagrams)^36^ (2025)	1	0.28	0.04	0.96	0.12	0.88
AlexNet OFDM-LMMSE (2D Constellation Diagrams)	1	1	0.99	1	0.96	0.94
VGG-19 OFDM-LMMSE (2D Constellation Diagrams)	1	1	0.7	1	0.68	0.93
ResNet50 OFDM-LMMSE (2D Constellation Diagrams)	1	1	1	1	0.95	1

Modulation Type	2FPK	4FQAM	8FPK	8FQAM	16FPK	32FQAM
AlexNet SC-LMMSE (3D Constellation Diagrams)	1	1	0.52	0.9	0.61	0.79
VGG-19 SC- LMMSE (3D Constellation Diagrams)	1	1	0.56	0.9	0.78	0.77
ResNet50 SC- LMMSE (3D Constellation Diagrams)	1	1	0.96	0.77	0.43	0.82
AlexNet OFDM-DFT, LMMSE (3D Constellation Diagrams)	1	1	0.93	1	0.99	1
VGG-19 OFDM-DFT, LMMSE (3D Constellation Diagrams)	1	1	0.98	1	0.99	0.77
ResNet50 OFDM-DFT, LMMSE (3D Constellation Diagrams)	1	1	0.97	1	0.94	0.98

**Table 7:** Comparison of AlexNet, VGG-19, and ResNet50 models using 2D and 3D constellation diagrams at 15 dB SNR.

Modulation type	BPSK	4QAM	8PSK	8QAM	16PSK	32QAM
AlexNet SC, LMMSE (2D Constellation Diagrams)^36^ (2025)	1	1	0.44	1	0.48	1
VGG-19 SC, LMMSE (2D Constellation Diagrams)^37^ (2025)	1	1	0.24	1	1	0.72
ResNet50 SC-LMMSE (2D Constellation Diagrams)^36^ (2025)	1	1	0.44	1	0.6	0.88
AlexNet OFDM-LMMSE (2D Constellation Diagrams)	1	1	1	1	1	0.97
VGG-19 OFDM-LMMSE (2D Constellation Diagrams)	1	1	0.94	1	0.96	0.95
ResNet50 OFDM-LMMSE (2D Constellation Diagrams)	1	1	1	1	1	1

Modulation Type	2FPK	4FQAM	8FPK	8FQAM	16FPK	32FQAM
AlexNet SC, LMMSE (3D Constellation Diagrams)	1	1	0.87	0.97	0.9	0.81
VGG-19 SC, LMMSE (3D Constellation Diagrams)	1	1	0.85	0.98	0.98	0.86
ResNet50 SC- LMMSE (3D Constellation Diagrams)	1	1	0.99	0.94	0.75	0.87
AlexNet OFDM-DFT, LMMSE (3D Constellation Diagrams)	1	1	0.95	1	1	1
VGG-19 OFDM-DFT, LMMSE (3D Constellation Diagrams)	1	1	0.99	1	1	0.86
ResNet50 OFDM-DFT, LMMSE (3D Constellation Diagrams)	1	1	0.98	1	0.96	0.98

According to Table 5, at 0 dB SNR, the modulation classification task becomes highly challenging because of severe noise and multipath propagation effects. For SC-LMMSE with 2D constellation diagrams^36^,^37^, reliable performance is mainly achieved for BPSK modulation, where AlexNet, VGG-19, and ResNet50 attain nearly-perfect accuracy. However, the classification accuracy for high-complexity modulation formats deteriorates significantly. For example, AlexNet achieves 0% for 4QAM and 0% for 16PSK, while VGG-19 and ResNet50 also exhibit weak classification capabilities for higher-order PSK and QAM modulations. These results highlight the limited discriminative capabilities of conventional 2D constellation representations under severe underwater channel conditions.

When the constellation representation is extended from 2D to 3D, while maintaining the same SC-LMMSE equalization framework, a noticeable improvement in classification performance is observed across all CNN architectures. Using 3D constellation diagrams, AlexNet, VGG-19, and ResNet50 demonstrate improved recognition accuracy for several modulation formats compared with their corresponding 2D-based counterparts. For example, AlexNet achieves 17% accuracy for 8FPK and 84% for 32FQAM, while VGG-19 achieves 23% for 8FPK and 80% for 32FQAM. Similarly, ResNet50 attains 98% for 8FPK and 77% for 32FQAM. These results confirm that the enhanced 3D signal-space mapping reduces constellation overlap between modulation classes and enhances modulation discrimination even under severe low-SNR conditions. Nevertheless, the performance for some higher-order FPK modulations remains limited at this SNR level.

The OFDM-DFT-LMMSE was also evaluated using the same 2D constellation representation. Compared with SC-LMMSE using 2D constellation diagrams, the OFDM-based framework significantly improves classification performance for most modulation formats across all CNN models. For instance, AlexNet improves from 0 to 92% for 4QAM and from 76% to 99% for 8QAM, while VGG-19 and ResNet50 also achieve substantial gains. These improvements demonstrate the effectiveness of OFDM-DFT-LMMSE in mitigating underwater channel impairments and improving constellation separability.

The highest classification performance is achieved when OFDM-DFT-LMMSE is combined with the proposed 3D constellation diagrams. Under this configuration, AlexNet, VGG-19, and ResNet50 achieve near-perfect classification accuracy for most modulation formats, including approximately 94–99% for 8FPK and nearly 100% for 8FQAM. These findings confirm that both the OFDM transmission framework and the three-dimensional modulation representation independently contribute to performance enhancement, while their integration provides the most robust classification capabilities under highly degraded underwater channels.

According to Table 6, at 10 dB SNR, overall classification accuracy improves noticeably across all evaluated schemes. Nevertheless, the comparative analysis still reveals clear differences between the impacts of constellation dimensionality and transmission frameworks. For SC-LMMSE with 2D constellation diagrams, AlexNet achieves only 40% accuracy for 8PSK and 12% for 16PSK, while VGG-19 and ResNet50 also experience noticeable degradation for high-order modulation schemes. When the same SC-LMMSE framework works on 3D constellation diagrams, the corresponding accuracies improve substantially across all CNN models. For example, AlexNet achieves 52% for 8FPK and 61% for 16FPK, while VGG-19 achieves 56% and 78%, respectively. In addition, ResNet50 achieves 96% for 8FPK, confirming the superior discriminative capabilities of the proposed 3D representation.

Similarly, applying OFDM-DFT-LMMSE while maintaining 2D constellation diagrams results in substantial performance improvements compared with SC-LMMSE using the same 2D representations. AlexNet achieves approximately 99% accuracy for 8PSK and 96% for 16PSK, while VGG-19 and ResNet50 also demonstrate significant gains. Further improvements are achieved when OFDM-DFT-LMMSE is combined with 3D constellation diagrams, where all evaluated CNN architectures consistently achieve classification accuracies above 93% for nearly all modulation formats. These observations verify that both OFDM processing and 3D constellation representation independently improve classification performance at moderate SNR levels.

According to Table 7, at 15 dB SNR, the overall classification performance further improves for all investigated configurations. For SC-LMMSE with 2D constellation diagrams, AlexNet, VGG-19, and ResNet50 achieve acceptable classification accuracies for most modulation formats, although some degradation persists for higher-order PSK signals. When 3D constellation diagrams are utilized within the same SC-LMMSE framework, the classification performance improves significantly across all CNN models. AlexNet, VGG-19, and ResNet50 achieve approximately 85–99% accuracies for most FPK and FQAM modulation formats, confirming the effectiveness of the proposed frequency-index constellation structure in enhancing constellation-space discrimination.

In addition, OFDM-DFT-LMMSE using 2D constellation diagrams achieves near-perfect classification performance for most modulation formats, highlighting the capabilities of OFDM processing to reduce underwater channel distortions and improve signal robustness. The best overall performance is once again achieved when OFDM-DFT-LMMSE is combined with 3D constellation diagrams, where classification accuracy approaches 100% across nearly all modulation formats for AlexNet, VGG-19, and ResNet50. These results demonstrate that the proposed three-dimensional modulation representation and the OFDM transmission framework each provides distinct and measurable performance improvements, while their combination delivers the highest modulation classification accuracy and robustness under challenging UWA channels.

## Conclusion

This paper presented a DL-based AMC framework for UWA communication systems using FPK and FQAM modulation schemes. The proposed framework depends on 3D constellation representations, the SC-LMMSE and the OFDM-DFT-LMMSE transmission schemes to improve modulation classification under severe underwater channel conditions. Simulation results showed that the proposed 3D constellation framework improved constellation separability and classification accuracy compared with conventional 2D representations, particularly under low-SNR conditions and for higher-order modulation formats. In addition, the OFDM-DFT-LMMSE framework consistently outperformed the SC-LMMSE framework across all investigated CNN models. The comparative evaluation using AlexNet, VGG-19, and ResNet50 confirmed the effectiveness of DL–based AMC in UWA environments, with deeper architectures generally providing more stable classification performance under challenging channel conditions. However, several limitations still exist in the current study. The proposed framework was evaluated using offline-generated datasets without experimental sea-trial validation, and we considered only SISO transmission with a limited set of modulation formats. Furthermore, the current framework relied mainly on constellation-image representations without incorporating complementary signal-domain features. In addition, a direct comparison with external UWA AMC methods was not feasible due to the lack of publicly available datasets and consistent experimental settings, which prevented a fair and reproducible evaluation. Although the adopted UWA-MACE channel model reflects realistic UWA propagation conditions derived from the MACE experiment, the present study is still based on simulation-generated data. Therefore, future work will focus on validation using real UWA measurements, advanced feature extraction, hybrid DL techniques, comparative investigations of transfer learning and random initialization strategies, and practical FPGA implementation for underwater communication systems.

## Data Availability

The datasets generated and/or analysed during the current study are available from the corresponding author upon reasonable request.
